# Preclinical evaluation of novel synthesised nanoparticles based on tyrosine poly(ester amide) for improved targeted pulmonary delivery

**DOI:** 10.1038/s41598-024-59588-1

**Published:** 2024-04-29

**Authors:** Eman Zmaily Dahmash, Nour Radwan Achkar, Dalia Khalil Ali, Qais Jarrar, Affiong Iyire, Shereen M. Assaf, Hamad Alyami

**Affiliations:** 1 Department of Chemistry and Pharmaceutical Sciences, School of Life Sciences, Pharmacy and Chemistry, Kingston University, London, KT1 2EE UK; 2 Department of Applied Pharmaceutical Sciences and Clinical Pharmacy, Faculty Pharmacy, Isra University, Amman, 11622 Jordan; 3 Department of Physiotherapy, Faculty of Allied Medical Sciences, Isra University, Amman, 11622 Jordan; 4 Aston Pharmacy School, College of Health & Life Sciences, Aston University, Birmingham, B4 7ET UK; 5 Department of Pharmaceutical Technology, Faculty of Pharmacy, Jordan University of Science and Technology, P. O. Box 3030, Irbid, 22110 Jordan; 6 Department of Pharmaceutics, College of Pharmacy, Najran University, 55461 Najran, Saudi Arabia

**Keywords:** Dry powder inhaler, Fluticasone propionate, Interfacial polycondensation, Salmeterol xinafoate, Tyrosine-based poly (ester amide), Drug delivery, Pharmaceutics

## Abstract

Fixed dose combinations (FDCs) incorporating two or three medicines in a single inhaler have been created to enhance patient compliance and hence clinical outcomes. However, the development of dry powder inhalers (DPIs), particularly for FDCs, faces challenges pertinent to formulation uniformity and reproducibility. Therefore, this project aimed to employ nanotechnology to develop a FDC of DPIs for market-leading medicines—fluticasone propionate (FP) and salmeterol xinafoate (SAL)—for asthma management. Nanoaggregates were prepared using a novel biocompatible and biodegradable poly(ester amide) based on the amino acid tyrosine, utilising a one-step interfacial polymerisation process. The produced tyrosine poly (ester amide) drug-loaded nanoparticles were evaluated for content uniformity, PSA, FTIR, TEM, DSC, XRD and aerodynamic performance (in vitro and in vivo). The optimised formulation demonstrated high entrapment efficiency– > 90%. The aerodynamic performance in terms of the emitted dose, fine particle fraction and respirable dose was superior to the carrier-based marketed product. In-vivo studies showed that FP (above the marketed formulation) and SAL reached the lungs of mice in a reproducible manner. These results highlight the superiority of novel FDC FP/SAL nanoparticles prepared via a one-step process, which can be used as a cost-effective and efficient method to alleviate the burden of asthma.

## Introduction

Recently, nanocomposite materials have been employed for targeted drug delivery (TDD) due to their capacity to carry sufficient concentrations of the active pharmaceutical ingredient (API) and release this directly to the target organ^1^. TDD systems offer various advantages such as reduced dosage, lower toxicity, enhanced bioavailability, longer release time, and site-specific controlled release^2^. For example, APIs targeting local or systemic effects can be administered via the pulmonary route. Polymers have played a significant role in the manufacture of TDD systems, by allowing controlled drug release of APIs across extended periods and customisable distribution of both moisture-sensitive and water-insoluble drugs. Modern TDD systems rely on rational polymer design that is tailored to a specific payload and devised to exert a variety of biological functions^3^.

The use of inhalable nanoparticles has generated considerable interest due to its ability to overcome the challenges encountered with conventional inhalable formulations in terms of low-dose delivery and insufficient targeting. Inhalable nanoparticles have the potential to reduce the required dose, improve solubility, enable targeted drug delivery, enhance absorption, and allow for pulmonary retention. This technology can ensure the effectiveness of medications in cases where the patient is unable to inhale effectively due to factors such as insufficient inspiratory flow rate, breath-holding problems, or difficulty coordinating the use of inhalation devices. As a result, inhalable nano-formulations show promise in the treatment of lung diseases such as COPD, asthma, lung cancer, COVID-19, and others^4,5^.

Lung tissues have powerful clearance mechanisms and physiological and anatomical barriers, which hinder the effectiveness of conventional dosage forms. Therefore, there is a pressing need for new classes of safe and effective TDD^6^. The clearance of particles by alveolar macrophages, which depends on particle size and surface charge, represents a significant barrier to achieving optimal extended drug release within the alveoli. Particles with sizes ranging from 1 to 5 μm are easily cleared by macrophages, while nanoparticles smaller than 200 nm cannot be recognised, particularly with negative surface charge^7^. To avoid macrophage uptake and prolong the residence time of drugs in the deep lung, inhalable nanoparticles based on size and electronegativity can be employed. This is optimal for the management of local disorders such as asthma. Furthermore, negatively charged or neutrally charged particles with high charge density are considered to possess superior mucus penetrability. Particles larger than 500 nm are trapped within the mucus fibre network, while those below 100 nm can more easily penetrate and traverse the mucus^8,9^.

Since their inception, biodegradable polymers have ushered in a new era in medical research, one that lasted for more than half a century and resulted in important advances in areas like medication delivery, biomaterials and tissue engineering as well as the creation of new medical devices^10^. Polymers were fundamental in the development of nano-drug delivery platforms, which were developed to overcome pharmacological constraints and surpass standard dosage forms. Nanoparticle (NP) drug delivery systems have been established because of research and breakthroughs in synthetic approaches, fabrication procedures, and statistical models for investigating the mechanisms of drug delivery systems^11,12^. Biodegradable polymeric NPs have demonstrated the ability to transport medications to target sites and overcome challenges with various barriers. One of the key benefits of adopting the NPs technique is that it can produce uniform formulations for potent or low-dose actives. This can be achieved when the API is encapsulated to produce uniform particles with a lower variation coefficient^13–15^.

A variety of biodegradable polymers and polysaccharides like agarose, alginate, carrageenan, hyaluronic acid, dextran, as well as cyclodextrins have been used in drug delivery due to the abundance of these substances in nature and their biocompatibility. However, despite their biodegradability, natural polymers demonstrate inter-batch variability which makes natural polymers not as attractive as synthetic polymers, which are versatile and varied in their characteristics^16–19^. Examples of synthetic polymers that have been employed for drug delivery systems include poly(esters), poly(ortho esters), polyanhydrides, and biodegradable polycarbonates^20^. Polymers commonly used for pulmonary drug administration need to possess properties such as safety, biodegradability as well as production of nanoparticles with sufficient charge (zeta potential) to avoid colloidal aggregation once settled in the lower parts of the respiratory system^21–23^.

Recently, amino acids have been imbibed in the synthesis of functional polymers. It is possible to produce a large range of well-defined functional amino acid/peptide-based optically active polymers using various polymerisation processes because of the vast variety of functional groups in amino acids^24,25^. Our previous work using amino acid-based polymers was reported^14,15,26,27^. For example, the amino acid (L-Lysine) was used in the development of nanoaggregates that encapsulated fluticasone propionate and salmeterol xinafoate for dry powder inhalers (DPIs) exhibited superior properties in terms of reproducible aerodynamic performance using lysine-based polyamide^15^.

Dry powder inhaler (DPI) formulations have several advantages over other pulmonary drug delivery formulations. DPI exhibits high stability during storage, is ideal for poorly water-soluble actives, can deliver higher doses compared to pressurized metered dose inhalers (pMDIs), is easy to transport and does not require patient coordination^28^. The development of DPIs is based on two key strategies: carrier-based formulation or aggregates. Carrier based DPI formulations entail the combination of micronized API, a carrier (mainly lactose), and an inhaler device. Despite the extensive use of carrier based DPIs, important drawbacks of this approach are poor content uniformity and reproducibility of aerodynamic behaviour of the formulations. The second approach is based on the use of aggregates, which are either consists of API alone (if the dose is high) or in combination with excipients. As the aggregates generate homogeneous particles, this approach eliminates the content uniformity and reproducibility concerns^29^.

However, the effective delivery of an API via the pulmonary route can be improved, particularly, for FDCs, by critically formulating the targeted API to produce desired attributes in terms of aerodynamic performance, particle size, shape charge and surface properties. Therefore, the aim of this study was to develop a DPI formulation for FDC of fluticasone propionate (FP) and salmeterol xinafoate (SAL) using nanoaggregates technology. The study investigated the synthesis of novel polymers made from the amino acid tyrosine and the cost-effective one step interfacial polycondensation technique for the simultaneous polymerisation and API encapsulation process, followed by in-vitro and in-vivo characterisation of the performance of the produced nanoparticles.

## Materials and methods

### Materials

Fluticasone propionate and salmeterol xinafoate powder were purchased from Discovery Fine Chemicals (Windborne, United Kingdom). Fisher Scientific supplied the dimethyl malonyl (Waltham, MA, USA). Methanol and dimethylformamide (DMF) were obtained from Tedia high purity solvent (Fairfield, USA). Alpha Chemika (Mumbai, India) supplied chloroform, acetonitrile HPLC grade, trifluoroacetic acid (TFA), and HPLC grade water. Diethyl ether, dimethyl sulfoxide (DMSO), potassium hydroxide (KOH), sodium hydroxide (NaOH), and sodium chloride (NaCl) were purchased from Labogens (Ahmedabad, Gujarat, India). Sigma (Pool, UK) provided the tyrosine amino acid and tetramethyl silane. All materials were used as supplied without any further purification.

### Synthesis of poly (ester amide) based on tyrosine (Tyr-PEA)

Interfacial polymerisation was employed to develop the Tyr-PEA^14,27^. Initially, 0.9 g (5 mmol) of tyrosine and 0.4 g of NaOH (10 mmol) were dissolved in 20 mL distilled water. The reaction mixture was cooled with ice, and 5 g of NaCl added. The organic phase was prepared by dissolving 0.85 g (5 mmol) of dimethyl malonyl in 10 mL of chloroform, which was thereafter added to the aqueous tyrosine-containing solution dropwise over 5 min under vigorous stirring at ambient room temperature. Stirring was maintained for an additional 30 min to complete the polymerisation process. The product was collected by suction filtration, washed three times with 5 mL portions of distilled water while filtrating and freeze-dried for 6 h to remove the residual solvent using benchtop freeze dryer (Virtis benchtop Pro with Omnitronics™ from SP Scientific (Warminster, PA, USA)).

### Synthesis of poly (ester amide) based on tyrosine-fluticasone propionate nanoparticles (FP-Tyr-PEA) and tyrosine-salmeterol xinafoate (SAL-Tyr-PEA) nanoparticles

The (FP-Tyr-PEA) and (SAL-Tyr-PEA) NPs were prepared using the same synthetic method employed for Tyr-PEA, however, the only change was that 10 mg of FP (or 2 mg of SAL) was added to the chloroform solution before the addition to the aqueous phase. Therefore, polymerisation and encapsulation were performed simultaneously^14,27^.

### Drug loading capacity

The final products of FP-Tyr-PEA NPs and SAL-Tyr-PEA NPs were initially passed through sieve with aperture size of 32 µm; and the resulting powders used to determine drug loading capacity for each API separately. For this, the amount of API within each product was quantified in two directions. The first was the entrapment efficiency (EE%) and the second was the loading capacity (LC), according to the method previously used by Alyami^15^ and the following equations:

1 $$ {\text{EE}} \left( \% \right) = \frac{{{\text{API}}_{t } - {\text{API}}_{f} }}{{{\text{API}}_{t} }} \times 100 $$

where the “${{\text{API}}}_{t}$” is total amount of API used in the synthesised formula, and “${{\text{API}}}_{f}$” is the quantity of API present in the filtrate (including the washing filtrate) and available in the liquid that was filtered after the polymers were formed (i.e., the amount of the API not entrapped within the produced product).

The LC was evaluated by quantifying the amount of API in the products (FP-Tyr-PEA) or (SAL-Tyr-PEA). That was carried out by accurately weighing 10 mg of each blend, which were then dissolved separately in 10 mL of methanol and analysed using the HPLC method. Samples were filtered using 0.45 µm syringe filter before analysis. The %LC was established using Eq. (2). ${{\text{FP}}/{\text{SAL}}}_{{\text{q}}}$ is the quantity of either FP or SAL quantified using the HPLC method in each blend. ${{\text{Blend}}}_{t}$ is the weight of the analysed sample (FP-Tyr-PEA or SAL-Tyr-PEA).

2 $$ {\text{LC}} \left( \% \right) = \frac{{{\text{API}}_{q} }}{{{\text{Blend}}_{t} }} \times 100 $$

### Preparation of FP-Tyr-PEA and SAL-Tyr-PEA NPs blend

Based on the results of EE and LC, the required final blend containing 250 µg of FP and 50 µg of SAL were prepared using geometrical blending technique. This blend of FP/Tyr-PEA and SAL/Tyr-PEA was made by weighing a ratio of 17 mg of FP/Tyr-PEA to 13 mg of SAL/Tyr-PEA. For example, to prepare 300 mg of the final blend, 170 mg of FP/Tyr-PEA was weighed and mixed with 130 mg of SAL/Tyr-PEA. The blend was mixed for 5 min using a Torbula^®^ 3D tumbler mixer (WAB AG, UK). The final blend was then passed through a 32 µm sieve and stored in an airtight container to be used within a week for further characterisation and analysis. For the NGI study, capsules were filled with 30 mg of the final FP/SAL/Tyr-PEA powder to ensure that each capsule provided 250 µg of FP and 50 µg SAL.

### Fluticasone propionate and salmeterol xinafoate analytical method validation

High performance Liquid Chromatography (HPLC) was applied for the quantitative analysis of FP and SAL, as previously described by^15^—but with modifications. A Dionex UltiMate^®^ 3000 (Waltham, MA, USA) HPLC system coupled with FORTIS HPLC column [5 µm, C18 (250*4.6 mm)] from Fortis Technologies Ltd, UK was used. The mobile phase comprised of acetonitrile: 0.125% TFA in water, in a ratio of (75:25, %v/v); and run time of 15 min. The UV detection wavelength was 239 nm for the two actives^15^, the column temperature was 20 °C, and the sample temperature was off. Sample injection volume was 20 μL, with flow rate of 1 mL/min.

The validation criteria included selectivity, linearity, limit of detection (LOD), limit of quantification (LOQ), precision, and accuracy; based on the International Conference on Harmonisation’s Q2 (R1) recommendations for the validation of analytical processes^30^.

### Characterisation of (FP/SAL-Tyr-PEA) NPs

#### Solubility test

To assess the solubility, 10 mg of each FP, SAL, Tyr-PEA, and (FP/SAL-Tyr-PEA) NPs powder was dissolved in 5 mL of different organic solvents (DMSO, methanol, diethyl ether, chloroform, acetonitrile, acetone and DMF) at room temperature for 48 h. Results were reported as soluble (+), insoluble (−), and slightly soluble (−/+) using visual observations.

#### Fourier-Transform infrared analysis (FTIR)

For FTIR (Perkin Elmer, OH, USA), a few milligrams of each sample were placed on a sample holder above the laser lens and secured with an adapter. Scans of the FTIR spectra were made with a resolution of 2 cm^−1^ and covered a wavelength range of 450–4000 cm^−1^.

#### NMR analysis

For the ^1^H-NMR and ^13^C-NMR measurements, tetramethylsilane (TMS) was used as an internal standard. The ^1^H NMR was carried out at 500 MHz while the ^13^C NMR was conducted at 125 MHz for the analysis of Tyr-PEA NPs. A Bruker Avance DPX 300 NMR spectrometer (125/500 MHz) spectrometer (Bruker DPX-500) from Bruker, (Massachusetts, USA) was used.

#### Differential scanning calorimetry analysis (DSC)

For DSC about 2 mg of each sample was placed in an aluminium pan and heated at a rate of 10 °C per minute till 300 °C. Nitrogen gas was purged at a rate of 50 mL/minute. A DSC Q200-TA instrument (TA Instruments, New Castle, DE, USA) was used for the analysis.

#### X-ray diffraction studies (XRD)

XRD analysis was conducted on samples equipped with a Cu Ka radiation source excitation voltage of 40 kV, and 15 mA current. Recordings were captured between 2θ ranging from 0° to 90° with a speed of 10°/min. The set sample was put on a glass holder and scanned in triplicate. OriginPro^®^ software was used for peaks analysis [OriginLab Corporation (Northampton, MA, USA)].

#### Swelling test

Accurately weighed 50 mg of the dry Tyr-PEA powder was placed in 15 mL distilled water and then shaken at 37 °C for various time intervals (0.5, 1, 2, 3, 4, 10, 24, and 48 h) (total 8 test tubes were used). The swollen Tyr-PEA was centrifuged at 1500 rpm for 5 min, and the water was filtered using filter paper from all tubes and then reweighed. The swelling capacity was calculated using the following formula:

3 $$ {\text{Swelling}}\;{\text{Capacity}} = \frac{{{\text{Mass}}\;{\text{of}}\;{\text{the}}\;{\text{Polymer}}\;{\text{after}}\;{\text{the}}\;{\text{test}} \left( {\text{g}} \right)}}{{{\text{Mass}}\;{\text{of}}\;{\text{the}}\;{\text{dry}}\;{\text{powder}} \left( {\text{g}} \right)}} $$

#### Content uniformity

50 mg of the FP-Tyr-PEA NPs and SAL-Tyr-PEA NP powders were accurately weighed and dissolved separately in 25 mL of methanol by vigorous shaking. After that, each solution was filtered using a syringe membrane filter 0.2 µm after 30 min in the bath sonicator. The HPLC method was used to assess FP or SAL content. Samples were made in triplicates and results were reported as mean ± SD.

#### In vitro release study

The method used to determine the release of FP and SAL from the FP-Try-PEA, SAL-Tyr-PEA nanoparticles, the physical mixtures and the marketed product was the dialysis membrane method, which is described in the literature^31^. First, 30 mg of FP-Try-PEA and SAL-Tyr-PEA were weighed individually and then redispersed in 5 mL of pH 7.4 phosphate buffer. Similarly, 30 mg of the physical mixture was prepared and dispersed in 5 mL of pH 7.4 phosphate buffer. Individual doses were also prepared from the marketed product as with the other formulations. These were then placed in dialysis bags (Sigma-Aldrich, Pool, UK) with a molecular weight cut-off of 14,000 Da. The bags were suspended in 30 mL of pH 7.4 phosphate buffer in covered beakers and kept in a shaking water bath maintained at 37 °C and 100 rpm. At different time intervals (0.5, 1, 2, 4, 6, 12, 24 h), 1 mL from the receiver compartment was taken out, and the APIs content was quantified using the HPLC method. Fresh buffer was added to maintain the sink conditions.

#### Particle size, polydispersity, and zeta potential analysis

Particle size, polydispersity and zeta potential of Tyr-PEA nanoparticles, FP, SAL, (FP/SAL-Tyr-PEA) NPs were evaluated using dynamic light scattering technique. A total of 5 mg of the material to be tested were suspended in 1 mL water. All measurements were made in triplicates and results were reported a mean ± SD.

#### Transmission electron microscopy (TEM)

TEM imaging of (FP/SAL-Tyr-PEA) NPs were captured. The process involved preparing a suspension of the sample (around 0.05 mg/mL) using deionised water as a suspending medium. Then about 10 μL of the prepared suspension was put on a copper grid and left for few minutes to allow the nanoparticles to settle on the grid. Then, the excess solvent was removed using filter paper and the sample was placed into TEM for imaging. Images were then processed using ImageJ software. TEM—Morgagni 268D; Fei Company (Hillsboro, OR, USA) was used to conduct the analysis. ImageJ software was used for the calculation of the average particle size.

#### Scanning electron microscopy (SEM)

The FP/SAL-Tyr-PEA NPs' surface morphology was examined using a scanning electron microscope (SEM) from Zeiss Evo50-Oxford instrument, UK. A few milligrams were scattered over an aluminium stub and coated with gold to aid electrical conduction. The sputter coater (Polaron SC7640, Watford, UK) was used with argon gas to achieve this. The collected micrographs were analysed using Smart SEM software from Zeiss Evo50-Oxford instrument, UK.

### In vitro assessment of the aerodynamic performance of (FP/SAL-Tyr-PEA) NPs

The next-generation impactor (NGI) Model 170 (Copley Scientific Limited, UK) was utilised for the assessment of both the in vitro deposition of FP and SAL and aerodynamic particle size distribution characterisation. Setting the pump flow rate at 60 L/min resulted in a flow of 4 L for almost four seconds. An Aerolizer^®^ inhalation device and a size 3 gelatine capsule (Pharmacare, Jordan) manually filled with 30 mg of (FP/SAL-Tyr-PEA) NPs, were used to assess FP and SAL aerosolisation performance. The weight of the blend in each capsule contained 250 µg and 50 µg of FP and SAL, respectively. Each test included six capsules to ensure accurate quantification of the APIs. FP and SAL content in each tray 1–7 and the MOC was obtained by dissolving the content of each tray in 10 mL of methanol. The trays/MOC were then placed in the sonication bath set at medium altitude without heat for 10 min to aid the dissolution of the two actives. After that, the liquid in each container, as well as the pre-separator, was transferred into volumetric flasks and volume adjusted with methanol. For HPLC analysis, samples were syringe filtered (0.2 µm membrane) and API content determined from calibration curves for both FP and SAL. A similar approach was employed for the assessment of a FP/SAL carrier based marketed FDC DPI formulation. The results obtained from the NGI analysis were used to calculate the main aerodynamic performance parameters of the final formulation blend. The total quantities of FP or SAL collected from stages that include induction tube, the pre-separator, trays 1–7 and the MOC were used to calculate the emitted dose (ED%) as can be seen from Eq. (4). ED is a true representation of the efficiency of the formulation to be discharged from the capsules. The next parameter was the respirable dosage (RD). It is the calculated form the total quantity of the API deposited on trays 2–7. This size range is favourable to DPI and it ensure deposition of the particles within the lower parts of the respiratory system^13,15,32^. The last aerodynamic parameters were the fine particle fraction of the emitted dose (FPF_ED_) and the fine particle fraction of the theoretical dose (FPF_TD_). The two parameters were calculated using Eqs. (5 and 6) respectively.

4 $$ {\text{ED}}(\% ) = \frac{{{\text{Cumulative}}\;{\text{API}}\;{\text{content}} \times 100}}{{{\text{Theoretical}}\;{\text{API}}\;{\text{Content}}}} $$

5 $$ {\text{FPF}}_{{{\text{ED}}}} \left( \% \right) = \frac{{{\text{RD}}}}{{{\text{ED}}}} \times 100 $$

6 $$ {\text{FPF}}_{{{\text{TD}}}} \left( \% \right) = \frac{{{\text{RD}}}}{{{\text{TD}}}} \times 100 $$

The developed formulation as well as a commercial dry powder inhaler, containing 250 µg/dose of FP and SAL 50 µg/dose, were evaluated for aerodynamic performance. Furthermore, the mass median aerodynamic diameter (MMAD) and the geometric standard deviation (GSD) were calculated using the deposition of DPI on various stages of the NGI using the USP method <601>^33^.

### In vivo study

A stainless-steel bent drencher needle of 0.9 mm diameter and 25 mm length from Socorex Isba SA (Vaud, Switzerland) was used for the in vivo study. The needle has a hollow stainless-steel tip with a 120°- bend that helped to keep the investigator's hand out of the line of sight and made it easy to view the epiglottis. The needle was connected to a 1 mL luer lock fitting syringe. The edge of the needle was smooth to ensure the animal's comfort and to avoid any potential injuries for the mice. The syringe was filled with 5 mg of (FP/SAL-Tyr-PEA) NPs formula, the marketed product or the Tyr-PEA alone.

The experiment included a total of 30 Swiss albino male mice, with an average weight ranging from twenty-five to thirty grams. The mice were retrieved from the animal house at Isra University, where they were being housed. The mice were housed in a setting that included temperatures between 22 and 25 °C, humidity levels between 67 and 77%, and a light/dark cycle that lasted for 12 h on and 12 h off. The animals were housed in a polypropylene cage measuring 30 cm by 22 cm by 16 cm, and they had unrestricted access to both standard feeding pellets and water. The animal handling and usage were approved by the Research Ethics Committee at Isra University and were aligned with established guidelines for the ethical treatment of animals in research. The mice were separated into three groups, with a total of six mice in each group, and given the FP and SAL mixture as detailed in Table 1.

**Table 1:** Distribution of mice groups within the in vivo study.

Groups	Description
Negative control (Blank polymer group (Tyr-PEA)) (one groups of 6 mice)	Each mouse within this group was exposed to 3 puffs of blank polymeric formula that contains the same constituents as the test formula but lacks the active ingredients
Positive control (Marketed Carrier-Based FP-SAL DPI Group) (one group of 6 mice)	Each mouse within this group was exposed to 3 puffs of marketed carrier-based FP-SAL DPI
Test group (FP-SAL-Tyr-PEA NPs Formula Group) (three group of 6 mice each)	Each mouse within this group was exposed to 3 puffs of FP-SAL-Tyr-PEA NPs formula (test formula)

Immediately after treatment, the mice were sacrificed by cervical dislocation, and the lungs were collected, weighted, and kept in the refrigerator at − 70 °C for further analysis.

#### Lung targeting study

Following the homogenisation of the lung tissues with an adequate volume of 1 mL of methanol using a homogeniser, the tissue homogenates underwent sonication. To ensure complete tissue breakdown, a diluted perchloric acid solution was prepared by mixing 1.195 mL of perchloric acid with 100 mL of distilled water. Subsequently, 75 µL of the diluted perchloric acid was added to 300 µL of the homogenates obtained earlier. The resulting mixtures were then centrifuged at 10,000 rpm for 10 min at 20 °C, and the supernatants were collected and stored at − 70 °C for HPLC analysis. Prior to injection into the HPLC device, the samples were diluted with 0.5 mL of acetonitrile and filtered using the same procedure employed for the validation of FP and SAL.

### Statistical analysis

All statistical analyses were performed using Minitab statistical pack version 18 for t-test and/or analysis of variance (ANOVA). Results were reported as mean ± standard deviation (SD) and relative standard deviation (RSD) as needed.

### Statement of animal rights

The in vivo study in this research complied with the ARRIVE guidelines and was carried out in accordance with the U.K. Animals (Scientific Procedures) Act, 1986 and associated guidelines, EU Directive 2010/63/EU for animal experiments with Scientific Research Ethics Committee Approval (SREC/08-43/2020/2021).

### Ethical approval and consent to participate

Approval was granted by the Scientific Research Ethics Committee at Isra university (SREC/08-43/2020/2021). This study does not involve any human subjects.

## Results and discussion

### Synthesis of poly (ester amide) based on tyrosine (Tyr-PEA)

**Figure 1:**
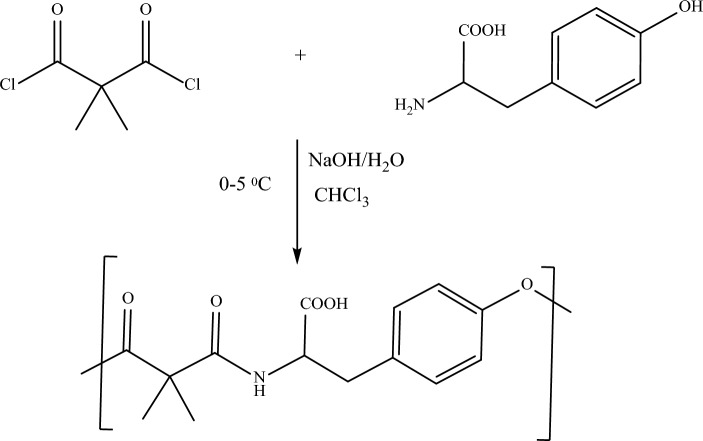
The synthesis of tyrosine-based poly (ester amide) using interfacial polycondensation technique.

Tyr-PEA was prepared using interfacial polycondensation from the reaction of dimethyl malonyl chloride with tyrosine amino acid. In this technique, the polymerisation process occurred at the interface between the aqueous layer containing tyrosine and the organic layer containing the dimethyl malonyl (Fig. 1). The vigorous stirring enabled the increase in the two layers of dispersion, where the interfaces are maximised due to the increase in the surface area available for the reaction. Moreover, the vigorous stirring facilitated the formation of fine particles of the polymer with reduced reactants and solvents encapsulation inside the polymer chain^15,32^. The interfacial polycondensation procedure was previously employed for the synthesis of FP and SAL polyamide nanoparticles based on L-lysine^15^.

### Synthesis of poly (ester amide) based on tyrosine- salmeterol xinafoate and fluticasone propionate nanoparticles (FP/SAL-Tyr-PEA) NPs

FP and SAL were encapsulated within the polymeric chain upon the polymerisation process. In this work, the produced poly (ester amide) nanoparticles (Tyr-PEA) were light-flowing powder containing FP and SAL for the delivery as a DPI by inhalation after sieving. The results of entrapment efficiency, loading capacity and overall process yield for FP and SAL are summarised in Table 2. The results were used to calculate the required quantity of each formula to make a single dose of the DPI containing 250 µg of FP and 50 µg of SAL; equivalent to 17.00 mg FP-Tyr-PEA NPs and 13 mg of SAL-Tyr-PEA NPs powder respectively. For effective production of DPI, a blend of the two polymeric formulations was prepared and 30 mg of the powder blend accurately weighted and added to each capsule. Content uniformity results of the produced blend showed high degree of uniformity that was 99.87 ± 2.05% (RSD = 2.5%) for FP and 95.01 ± 3.97% (RSD = 4.18%) for SAL. Those results were based on 10 samples taken randomly from three batches.

**Table 2:** Summary of entrapment efficiency (EE%) Loading capacity (LC%) and process yield (PY%) of FP and SAL from the corresponding FP-Tyr-PEA NPs and SAL-Tyr-PEA NPs (mean ± SD, n = 3).

API	EE%	LC%	PY%
FP	93.93 ± 5.68	1.45 ± 0.23	89.6 ± 5.2
SAL	95.48 ± 6.87	0.39 ± 0.06	91.1 ± 7.3

### HPLC method for the concurrent quantification of FP and SAL

**Figure 2:**
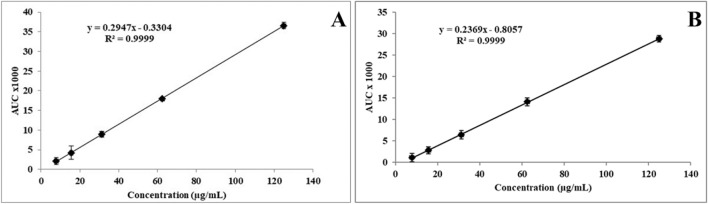
Beer-Lambert’s calibration curve of FP (**A**) and SAL (**B**) ranging from 7.81 to 125 µg\mL (mean ± SD), n = 3 measured at 239 nm.

The analytical method was based on a previously validated method^15^. However, the current formulation was based on a different amino acid. The next few sections highlight the validation process based on ICH guidelines. Initially the method was tested for its specificity to the active ingredients (FP and SAL) where the focus was to ensure no overlapping in HPLC chromatograms and specific peaks that corresponds to the actives as well as the polymer. The Tyr-PA, FP and SAL were tested separately, and the results demonstrated the specificity of the used method for concurrent measurement of FP and SAL that lacks any interference from the polymer (Tyr-PEA). The retention time for FP was 5.9 ± 0.22 min, whereas SAL eluted at 4.5 ± 0.17 min. There was no characteristic peak for the amino acid within this range. Calibration curves for FP or SAL concentrations that ranged from 7.81 to 125 µg/mL were constructed (Fig. 2) where the two actives demonstrated a good linearity over the specified concentration range.

Recovery experiments were done to determine the intraday repeatability and reproducibility (inter-day) as an indication of the method's accuracy. The results are shown in Table 3, which demonstrated good repeatability and reproducibility as evident from the low RSD results that did not exceed 2.65%. To ensure equipment precision one concertation was repeated ten times and the recovery as well and RSD were calculated for FP and SAL. Results are depicted in Table 3. The results revealed a precise method as the RSD was below 2%^30^. The limit of detection (LOD) and limit of quantification (LOQ) results for FP and SAL are shown in Table 3. Overall, the results showed an optimised and validated method for concurrent quantification of FP and SAL.

**Table 3:** Validation parameters (precision, reproducibility, inter and intraday reproducibility, LOD and LOQ) of FP and SAL HPLC method.

	Intraday % Recovery (mean ± SD) (n = 3)	
Concentration (µg/mL)	FP	SAL
125	99.87 ± 2.15	99.12 ± 1.65
62.5	101.07 ± 1.14	99.28 ± 2.01
31.25	97.65 ± 2.05	102.05 ± 2.65
	Inter-day % Recovery (mean ± SD) (n = 9) (three days)	
125	96.25 ± 2.08	98.55 ± 3.04
62.5	99.97 ± 4.09	98.43 ± 3.97
31.25	98.76 ± 4.66	96.54 ± 4.76
Precision mean ± SD, (RSD), n = 10	FP	SAL
62.5 (µg/mL)	101.78 ± 1.95 (1.92%)	99.12 ± 1.02 (1.03%)
LOD (µg/mL)	1.38	1.89
LOQ (µg/mL)	4.18	5.72

### Characterisation of Tyr-PEA and (FP/SAL Tyr-PEA) NPs

#### Solubility test

The purpose of assessing the solubility of the active ingredients, the polymer and the formula was to determine the solubility of these components in various organic solvents. This information is useful for conducting different analytical techniques such as HPLC and NMR. The solubility of FP, SAL, Tyr-PA, and (FP/SAL-Tyr-PEA) NPs were tested in different common organic solvents. The results are summarised in Table 4. FP, SAL, Tyr-PEA and (FP/SAL-Tyr-PEA) NPs were found to be soluble in methanol, DMSO and DMF. Therefore, methanol was chosen for HPLC analysis of both FP and SAL, while DMSO was used for the NMR study.

**Table 4:** Solubility test ( +) means soluble at room temperature, ( −) means insoluble at room temperature, (+ /−) means slightly soluble measured at 25 °C.

	DMSO	Methanol	Diethyl ether	Chloroform	Acetonitrile	Acetone	DMF
FP	+	+	−	+	+	+ /-	+
SAL	+	+	+ /-	+	−	+ /-	+
Tyr-PA	+	+	−	+ /-	−	−	+
(FP/SAL Tyr-PEA) NPs	+	+	−	+ /−	−	−	+

#### FTIR analysis

**Figure 3:**
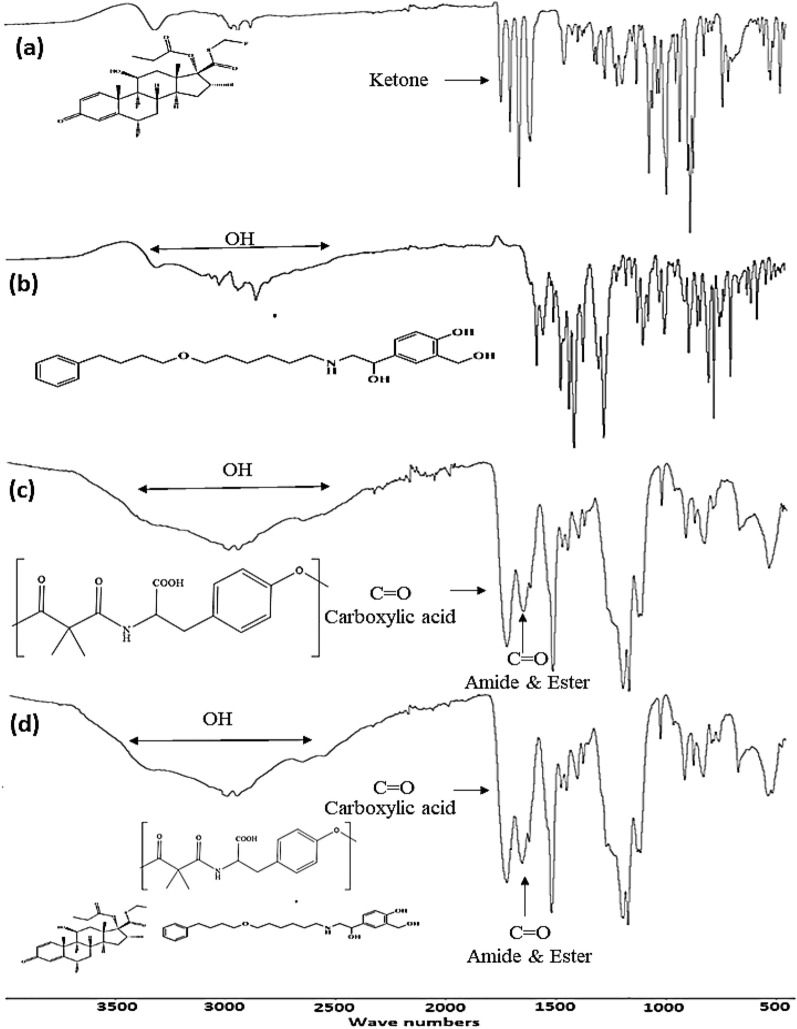
FTIR spectra of (**A**) FP (**B**) SAL (**C**) Tyr-PEA (**D**) FP-Sal Loaded Tyr-PEA NPs over a wavenumber range of 450–4000 cm^−1^.

As can be seen from Fig. 3, the FTIR spectra indicated typical absorption bands for all organic functional groups that are present in FP, SAL, Tyr-PEA and (FP/Sal-Tyr-PEA) NPs. The FTIR spectrum of FP showed three characteristic sharp bands that represent (C=O) groups. These bands appeared at 1744 cm^−1^, which corresponds to the ester carbonyl group; 1701 cm^−1^, which corresponds to the thioester carbonyl; and 1661 cm^−1^, which corresponds to the ketone carbonyl bond. The FTIR spectrum of SAL, revealed characteristically sharp bands at 3319 cm^−1^ for the OH band and 3024 cm^−1^ for the NH band. The amide group is associated with three characteristic infrared stretching absorptions. Stretching vibrations of the amide carbonyl bond appeared at 1644 cm^−1^, and the NH bond of the amide group was observed in 3263 cm^−1^and 1514 cm^−1^ for stretching and bending vibrations. The ester carbonyl was observed at 1635 cm^-1^. A strong band for the carbonyl bonds stretching vibrations for carboxylic carbonyl at 1715 cm^−1^. The carboxylic acid O–H showed stretching vibrations as a very broad band from 3462 to 2570 cm^−1^. The comparison between (Tyr-PEA) and (FP/SAL-Tyr-PEA) NPs FTIR spectra showed the same characteristic bands. This may be due to the low loading of FP and SAL as well as the overlapping between the polymer and drug bands, as shown in Fig. 3.

#### NMR analysis

**Figure 4:**
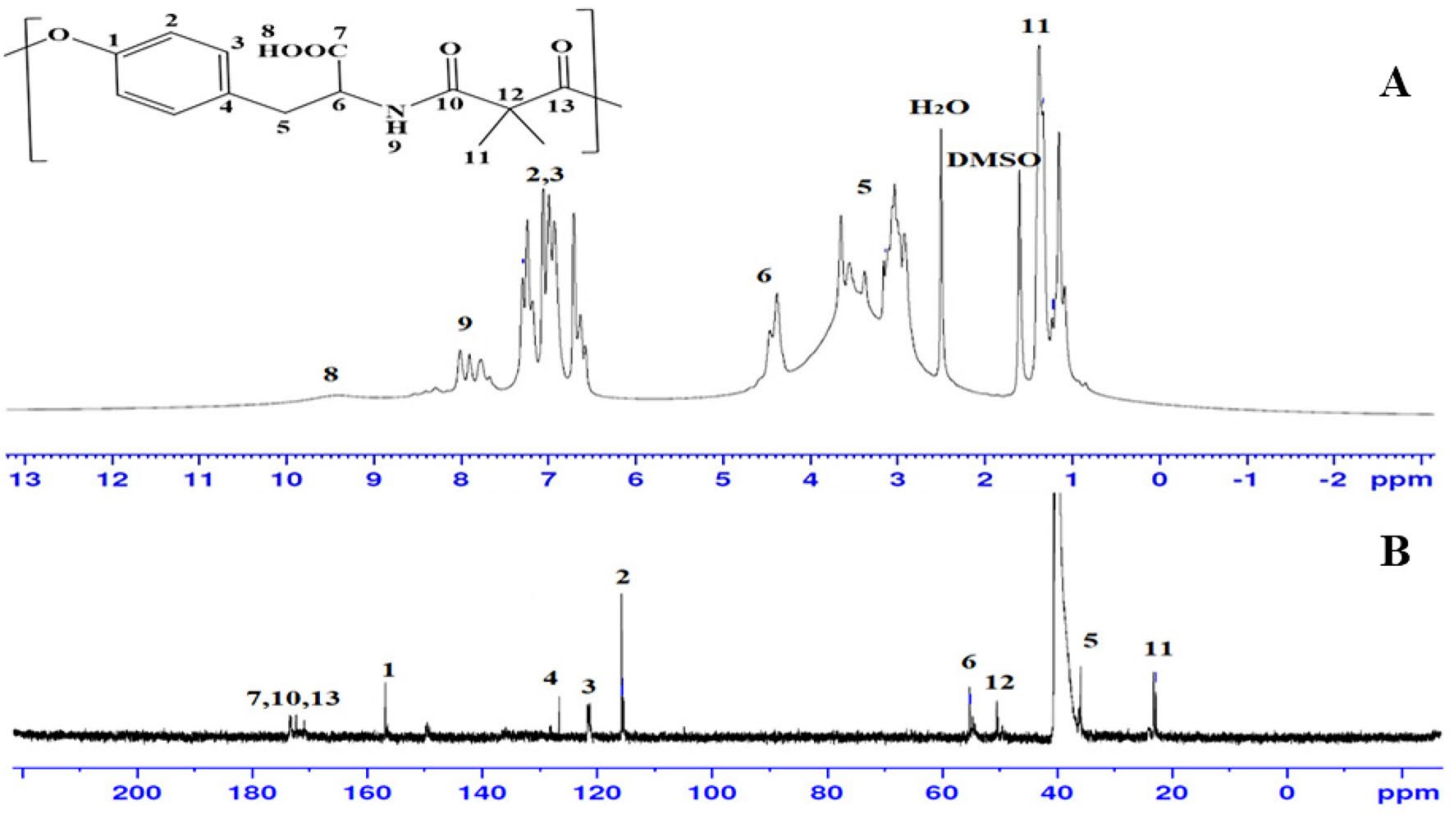
NMR analysis for Tyr-PEA to confirm the chemical structure of the polymer (**A**) 1H-NMR and (**B**) ^13^C-NMR spectroscopy.

The Tyr-PEA was also analysed by ^1^H-NMR and ^13^C-NMR spectroscopy to confirm their chemical structure (Fig. 4). The ^1^H-NMR spectrum of the synthesised Tyr-PEA confirmed their proposed structures; a broad peak appeared at 9.44 ppm due to the proton of the carboxylic acid group (–OH) (Fig. 4A) and doublets peak appeared at 7.90 ppm due to the proton of the amide group (–NH), multiplet peaks appeared at range 6.66–7.31 ppm that were assigned to protons of the aromatic ring of tyrosine unit. The signal for the protons of the (–CH_2_) group attached to the aromatic ring (Ar–CH_2_) appeared as a doublet at 3.51 and 3.01 ppm. The signal of the (–CH) proton appeared as a triblet at 4.37 ppm. The protons of the CH_3_ of the malonate unit appeared at 1.25 ppm.

The structure of the polymer was also confirmed by ^13^C-NMR (Fig. 4B). The most significant feature in the ^13^C-NMR was the characteristic peaks of the formed ester carbonyl bond at 172.3 ppm and amide carbonyl at 171.1 ppm compound. The carbons of the malonate carbons unit were observed at 55.2 and 15.6 ppm. The benzene carbons in the tyrosine unit were observed at the range 115.4, 126.6, 130.8, and 156.4.1 ppm.

#### XRD analysis

**Figure 5:**
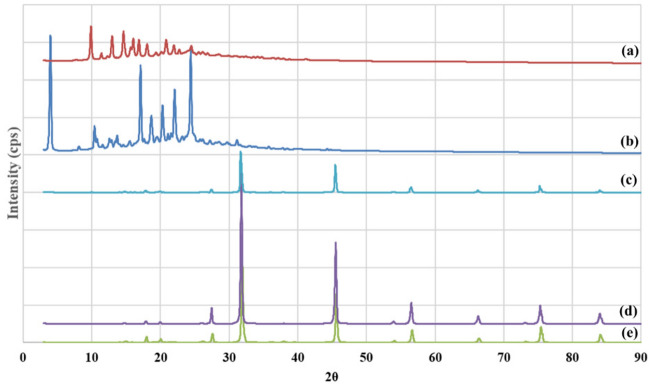
Powder XRD patterns: (**a**) FP, (**b**) SAL, (**c**) (FP/SAL-Tyr-PEA) physical Mixture (**d**) (FP/SAL-Tyr-PEA) NPs and (**e**) Tyr-PA.

XRD analysis of FP, SAL, Tyr-PA, (FP/SAL-Tyr-PEA) NPs as well as a physical mixture (containing similar SAL and FP concentrations as the formulations) of the actives and the polymer are depicted in Fig. 5. The XRD patterns of the two actives (FP and SAL—Fig. 5a and b respectively) revealed sharp peaks, depicting the crystalline nature of the two materials. Similar results have been reported earlier^15,27^. The Tyr-PEA diffractions revealed some features of crystallinity with characteristic peaks at 27.5°, 31.7°, 45.5°, 56.6°, and 75.3°. The same peaks were evident in both the physical mixture and the API loaded Tyr-PEA NPs. The API containing formulations (the physical mix and the FP/SAL-Tyr-PEA NPs) did not reveal any peaks that are related to the APIs (FP and SAL). This was attributed to the very low content of FP and SAL within the two products; therefore, it was not possible to evaluate any change in the crystallinity of FP and SAL.

#### DSC

**Figure 6:**
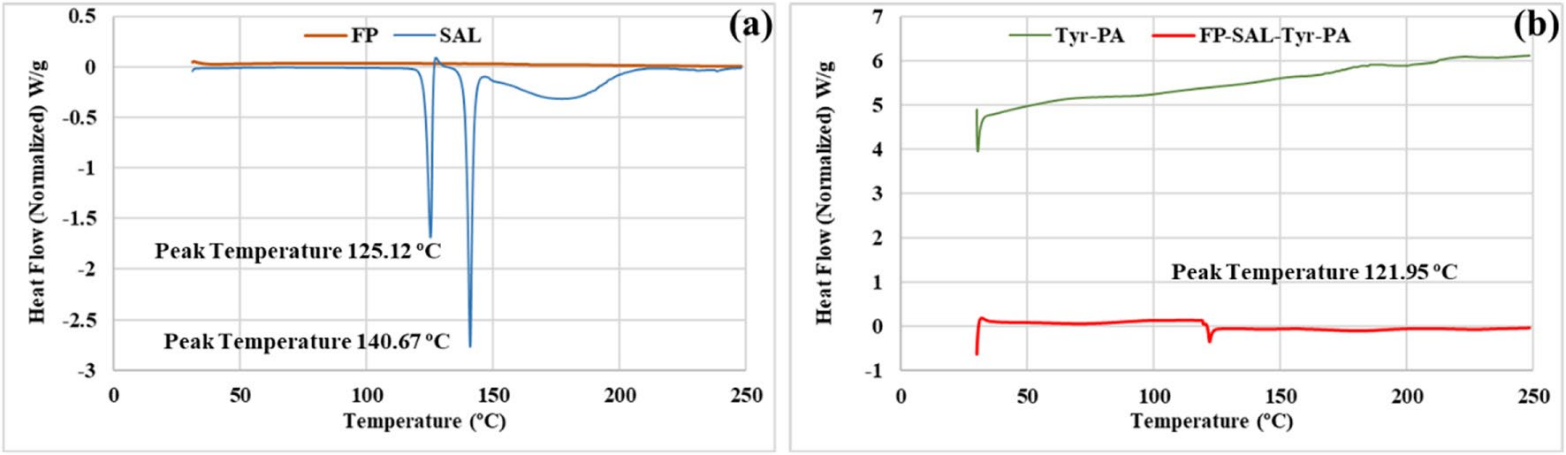
DSC thermograms for (**a**) FP and SAL, (**b**) Tyr-PEA and (FP/SAL-Tyr-PEA) NPs.

**Figure 7:**
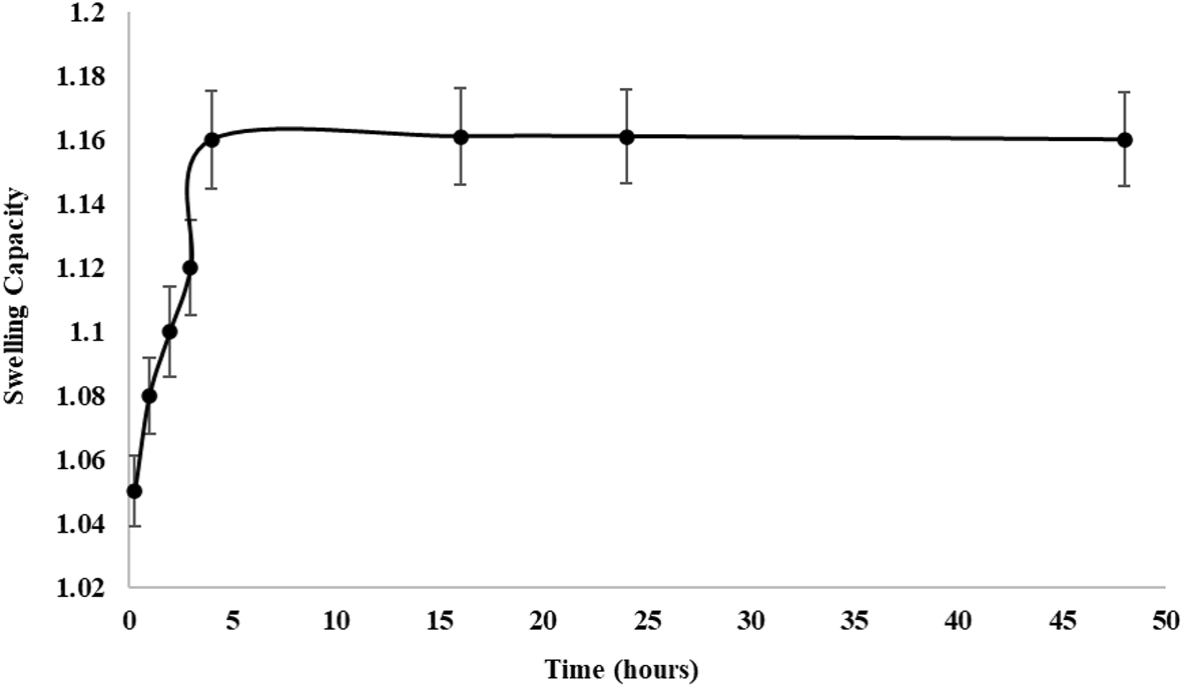
Swelling capacity ratio of Tyr-PEA over time (mean ± SD, n = 3).

**Figure 8:**
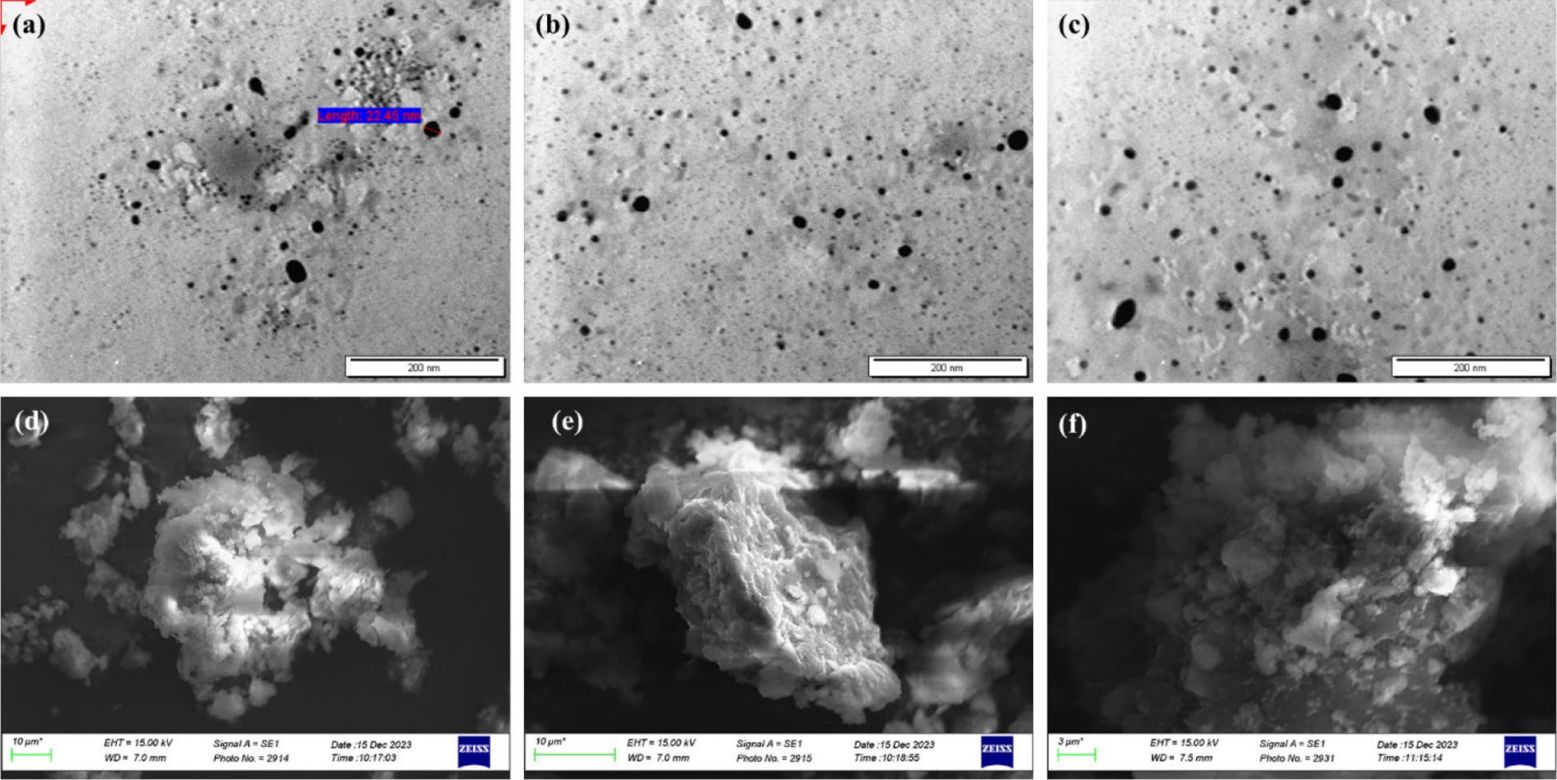
**(a–c)** TEM micrographs of (FP/SAL-Tyr-PEA) NPs highlighting the particle shape and size Scale bar = 200 nm, (d-f) SEM micrographs of (FP/SAL-Tyr-PEA) NP highlighting the aggregated nature of the NPs.

DSC analysis demonstrated the thermal behaviour of the used materials (Figs. 6 and 7). As reported earlier (Alyami et al. 2022), SAL demonstrated two endothermic peaks at 125.12 and 140.67 °C which was attributed to the presence of two crystal forms of the crystalline material^15,34^. As for FP, the reported melting point of 261–273 °C^15^ was beyond the measurement range of the equipment, therefore, this could not be identified. Figure 8b shows the thermograms of Tyr-PEA and the (FP/SAL-Tyr-PEA) NPs. The polymer did not show any characteristic endothermic peak despite being semi crystalline in nature, and there was no clear endothermic peak within the used range. Interestingly, the DSC thermogram of the formulation showed a similar trend to the polymer alone, with a small endothermic peak at 121.95 °C that represents the presence of SAL. This suggests that SAL may have retained its crystalline nature. The lower enthalpy was attributed to the very low content of the API within the polymer.

#### Swelling test

Figure 7 presents the results of the degree of swelling exhibited by Tyr-PEA at various time points. In general, the swelling capacity increased till 5 h after which there was no further increase in water intake. Overall, the swelling capability of the polymer was moderate to low, and this is favourable for pulmonary drug delivery. After one hour, the polymer swelling did not exceed 8%. Upon inhalation, particles are subject to humid environment and if the swelling capacity is high, the particles might deposit on the upper parts of respiratory system, which is not favourable. Effective DPI should successfully deliver the active ingredient into the alveolar region. A study reported a swelling of inhalable swellable polymer to exceed 100 times the previous weight^35^. It is expected that there would be no drastic increase in particle size upon inhalation, due to low polymer swelling capacity and therefore, favourable property of the formulation in terms of aerosolisation behaviour.

#### Particle size, zeta potential TEM and SEM analysis

The TEM micrographs of the produced NPs loaded with FP and SAL are presented in Fig. 8. As can be seen, the particles were within the nanosized range. Using Image J software, the average particle size was 21.9 ± 4.5 nm (n = 45). Particles were spherical in shape. However, the average particle size as obtained from laser diffraction was 105.32 ± 20.05 nm. This difference in size using the two methods could be attributed to the nature of the polymer which upon hydration, showed an increase in size during laser diffraction analysis, whereas the TEM revealed the actual size of the NPs. Such phenomena were previously reported in various publications, where during laser diffraction the presence of a hydrodynamic layer around the polymer could affect the size and hence a larger particle size was observed^36^. Further, it is possible that the particles were aggregating, (polydispersity index (PDI) was 0.337) during the laser diffraction analysis. Similar results have been reported in other research^32^. SEM micrographs were captured to examine the morphology and surface characteristics of NP aggregates^51^. The NPs had irregular shapes and rough surfaces, as indicated by the images (Fig. 8d–f). The SEM images revealed the aggregated nature of the NPs, with an irregular surface that showed deposition of fine particles on it. These depositions are likely to be the SAL and FP particles.

The zeta potential for the produced particles was in the negative scale (− 37.32 ± 3.48 mV) such results are promising as it can aid colloidal de-aggregation of the particles due to repulsive forces between particles. Research results noted that when a zeta potential is above + 30 or below − 30, repulsive forces will be effective and stable nanoparticles would be observed (Joseph et al. 2019). The negative charge is attributed to the carboxylic acid group within the polymer.

### In vitro assessment of the aerodynamic performance of (FP/SAL-Tyr-PEA) NPs

**Figure 9:**
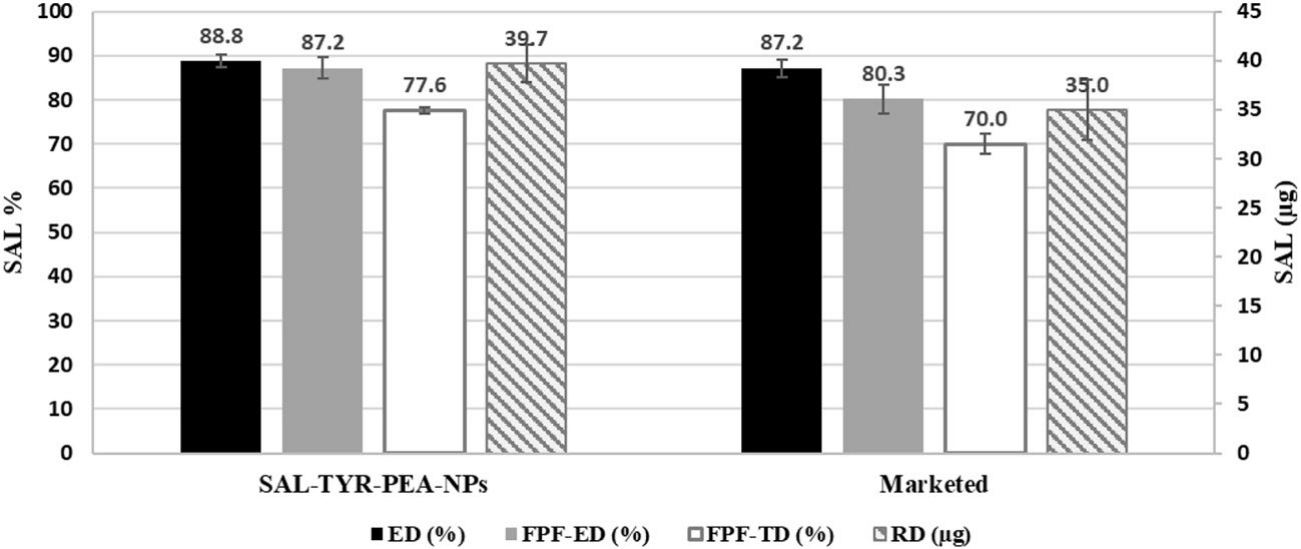
Aerodynamic parameters of SAL from FP-SAL-Tyr-PEA NPs, presenting the key parameters for SAL. ED: emitted dose; FPF-ED: fine particle fraction of emitted dose; FPF-TD: fine particle fraction of theoretical dose; RD: respirable dose. Results are presented as mean ± SD, n = 3.

**Figure 10:**
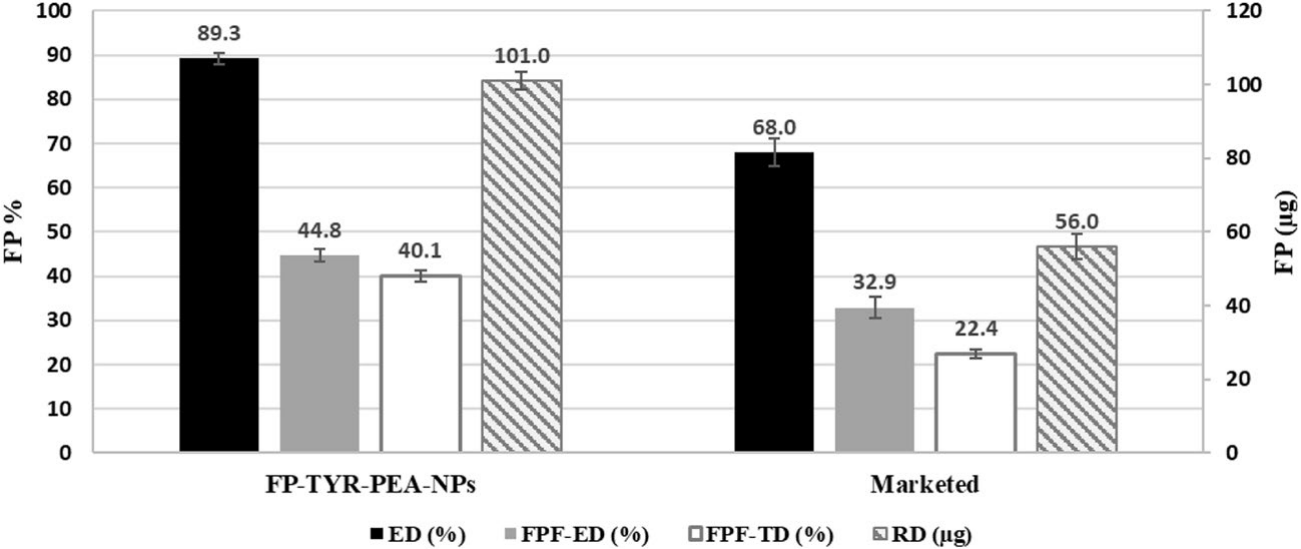
Aerodynamic parameters of FP from FP-SAL-Tyr- PEA NPs, presenting the key parameters for FP. ED: emitted dose; FPF-ED: fine particle fraction of emitted dose; FPF-TD: fine particle fraction of theoretical dose; RD: respirable dose. Results are presented as mean ± SD, n = 3.

NGI analysis was used to evaluate the aerodynamic performance of SAL and FP nanoparticles and results were compared with the carrier-based marketed DFC DPI product of fixed-dose combination of the two actives. Results of SAL and FP aerodynamic performance are summarised in Figs. 9 and 10, respectively. %ED, %FPF_ED_, RD and the FPF_TD_ were evaluated.

The emitted dose (ED) is known as the percentage of the theoretical dose that is emitted from the capsules upon actuation^14^. The results in Fig. 9 revealed that the prepared API-loaded NPs were able to successfully produce 88.8% of the available theoretical dose (50 µg/ puff) for SAL, which was comparable to the marketed product (87.2%). The difference between the produced formula and the marketed product for SAL was not statistically different (t-test, *p* = 0.42).

Regarding the respirable dose, which represents the amount of the API that reached the lower parts of the respiratory system, (RD), SAL-Tyr-PEA NPs showed higher RD (39.7 µg) per actuation than the marketed product (35 µg) per actuation, however, the difference was statistically non-significant (t-test, *p* = 0.248). Therefore, the produced formula for SAL, resulted in a comparable result with the marketed product for the delivery of SAL.

The FPF_ED_ represents the percentage of the emitted dose that can reach the lower parts of the respiratory system and has aerodynamic particle size between 1 and 5 µm^37^. The FPF_ED_ for SAL released from the produced NPs was 87.2% which was comparable with the marketed product at 80.3% (t-test, *p* = 0.143). The FPF_TD_ represents the percentage of the available theoretical dose that is within the range of 1–5 µm and will reach the lower parts of the respiratory system. When compared with the marketed product, our formula produced higher levels of FPF_TD_ (*p* = 0.028).

The deposition pattern of FP from FP-SAL-Tyr-PEA NPs are depicted in Fig. 10. The ED demonstrated a significantly higher (t-test *p* < 0.05) level of 89.3% comparable with the marketed product (68%). Similarly, the RD of FP from our NPs showed a statistically significant difference when compared to the marketed product. Almost double the quantity was within the size of 1–5 µm (t-test *p* = 0.01). The other two parameters in terms of FPF_ED_ and FPF_TD_ were higher for our formulation for FP when compared to the marketed product (t-test, *p* < 0.05 for both). The results of our work confirmed the superiority of our formulation in delivering higher percentages of FP to the lower parts of the respiratory system. It is worth mentioning that the test was repeated three times, and the low SD of the aerodynamic parameters confirmed the reproducibility of the formulation.

In general, the results of the aerodynamic performance of the synthesised FP/SAL- Tyr-PEA NPs formulations containing tyrosine as an amino acid highlighted the superiority of the nanoaggregates in delivering particles to the lungs. The low SD of the aerodynamic parameters indicated the reproducibility of results. Table 5 presents the MMAD and GSD values for two products. The MMAD values indicate that the particle sizes fall within the optimal inhalable range of 1–5 µm. The smaller the MMAD value, the higher the probability of particles depositing in the lung/alveolar region. However, there is a slight but significant difference between the FP MMAD values of the two products. The GSD values suggest that both products have a narrow particle size distribution, but again, there is a statistically significant difference between the two products in terms of GSD. In the case of nanoparticles, once the micro-scale particles deposit in the lung, they tend to disintegrate into smaller nanosized particles, as confirmed by the TEM and PSA results.

**Table 5:** Comparison between MMAD and GSD between the (FP-SAL/Tyr-PA) NCs and the marketed (FP-SAL) DPI carrier-based product (mean ± SD, n = 3).

Formulation	API	MMAD	*P*	GSD	*P*
SAL-FP-Tyr-PEA	SAL	1.598 ± 0.027	0.0075	0.054 ± 0.004	0.0019
Marketed	SAL	1.524 ± 0.017		0.079 ± 0.005	
SAL-FP-Tyr-PEA	FP	1.687 ± 0.024	0.0028	0.068	0.0014
Marketed	FP	1.441 ± 0.051		0.082	

### Release studies

**Figure 11:**
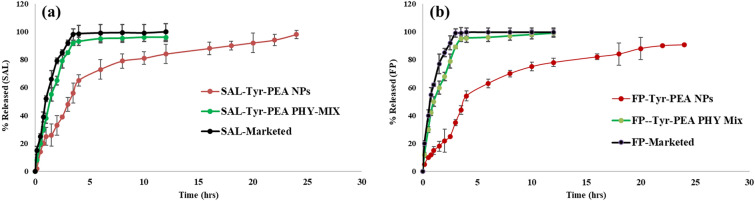
Release profile for (**a**) SAL over 24 h from SAL-Tyr-PEA NPs, SAL & Tyr-PEA Physical mixture, and the marketed product (**b**) FP over 24 h from FP-Tyr-PEA NPs, FP & Tyr-PEA Physical mixture, and the marketed product (mean ± SD, n = 3) [original FP content is 250 µg and 50 µg per 30 mg of FP/SAL-Tyr-PEA NPs and physical mix] using phosphate buffer pH 7.4.

The release profile of the two actives from the final FP/SAL-Tyr-PEA NPs exhibited an extended-release pattern (Fig. 11). Apparently, after 12 h, FP showed a release of 78.07 ± 3.6% whereas SAL release reached almost 84 ± 6.9% during this period. It is clear from the release profile that the prepared formula demonstrated an initial burst effect within the first hour of 15.13% for FP, and 24.89% for SAL. This could be attributed to the API particles attracted loosely to the surface of the polymer which was followed by a sustained release afterwards. It is worth noting that the concentration of the two actives in the liquid did not exceed the saturation solubility and hence sink conditions were maintained. In addition, the release profile of the two active ingredients from the physical mixture and the marketed product showed that over 80% of FP and SAL were released within 2 h. There was a slight delay in the release process due to the diffusion process through the dialysis bag. The release pattern of the marketed product was somewhat faster than the physical mixture, however, the difference was not significant (*p* > 0.05).

### In vivo study

**Figure 12:**
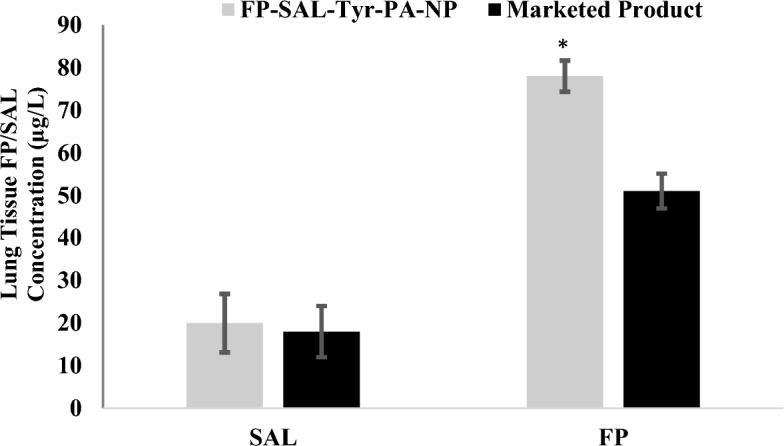
FP/SAL content in the lung’s mice (mean ± SD, n = 7) for FP-SAL-Tyr-PEA NPs and the marketed Product. (*) indicates a significance difference compared to the marketed product using a t-test.

Data from mice treated with blank polymer showed no detectable drug deposition in the lungs. In contrast, both the FP-SAL-Tyr-PEA NPs and the commercially available inhaler exhibited detectable drug levels as shown in Fig. 12. The FP-SAL-Tyr-PEA NPs demonstrated notably superior pulmonary delivery of both FP and Salmeterol SAL compared to the marketed formulation. Specifically, the FP-SAL-Tyr-PEA NPs achieved a significantly (t-test, *p* = 0.028) higher concentration of FP than the commercial counterpart, emphasising its efficacy in facilitating FP delivery. However, there was no significant difference in SAL concentrations between the two inhalers. These findings indicate the promising therapeutic potential of the FP-SAL-Tyr-PEA NPs, particularly in optimising FP delivery for enhanced asthma management.

## Conclusions

Formulation development of DPIs with excellent content uniformity and reproducible aerodynamic performance are challenging particularly for FDC. Therefore, the aim of this project was to develop a FDC of FP and SAL, which was loaded on a novel tyrosine-based poly(ester amide). Using interfacial polycondensation approach, polymerisation and API encapsulation were completed in a one step process. This resulted in nanoparticles with an average particle size of was 21.9 ± 4.5 nm. The produced formulation was able to deliver high emitted dose for FP (89.3%) and SAL (88.8%) that were higher than that of the carrier based marketed product (87.2% for SAL and 68% for FP). Molecular and surface characterisation studies supported the production of the polymer. The animal study findings confirmed the delivery of FP/SAL-Tyr-PEA NPs to the lower parts of the respiratory system that was similar for SAL but superior for FP than the carrier based marketed product. Overall, this work developed and delivered novel FP and SAL nanoaggregates to the lung and therefore, has a potential to act locally in the targeted area over an extended period. This also illustrated a simple, cost-effective process as an alternative to the carrier-based formulations for DPIs.

## Data Availability

All data generated or analysed during this study are included in this published article.
